# Editorial: Loss of taste and smell in COVID-19 patients: A prognostic tool and a starting point to investigate the action of SARS-CoV-2 in the central nervous system

**DOI:** 10.3389/fncel.2023.1191227

**Published:** 2023-04-03

**Authors:** Margherita Maffei, Andrea Mazzatenta, Nicola Origlia

**Affiliations:** ^1^Department of Biomedical Sciences, Institute of Clinical Physiology, National Research Council (CNR), Pisa, Italy; ^2^Department of Neuroscience, Imaging and Clinical Science, University “G. d'Annunzio”, Chieti-Pescara, Italy; ^3^Department of Biomedical Sciences, Institute of Neuroscience, National Research Council (CNR), Pisa, Italy

**Keywords:** anosmia, ageusia, brain, long COVID, prognosis, ACE2

When this topic was launched the presence of COVID-19 central nervous system related symptoms was just emerging. Indeed, the focus on COVID-19 symptoms in the first half of 2020 was primarily on respiratory symptoms such as cough, fever, and difficulty breathing. However, as more data was collected, it became evident that COVID-19 could affect multiple organ systems, including the central nervous system. The observation of anosmia and ageusia in COVID-19 patients was initially considered anecdotal, but subsequent studies confirmed that these symptoms were indeed common among COVID-19 patients. The exact biological mechanisms behind these symptoms are still being investigated, but it is believed that they may be related to the virus ability to invade and damage the cells of the olfactory and gustatory systems and, through those neural routes, the rest of the brain. Nowadays the long-term effects of COVID-19 are a major concern, particularly in terms of cognitive function. Many patients, who have recovered from COVID-19 continue to experience symptoms such as brain fog, thinking and memory problems. The exact duration of these symptoms is still unknown, but ongoing research is providing insights into the biological mechanisms behind the neurocognitive aspects of “long COVID”. In this context it is worth mentioning a longitudinal study recently appeared in Nature (Douaud et al., 2022) in which the brain of hundreds of participants from the UK Biobank was imaged by magnetic resonance before and after COVID-19 disease. By using automated, objective, and quantitative methods, they obtain a consistent spatial pattern of longitudinal abnormalities in limbic brain regions forming a mainly olfactory network associated to significant cognitive decline. An interesting issue raised by the authors for future investigations pertains the possibility of a potential future vulnerability of the limbic system in affected individuals with implications for memory impairment.

A first effort in clinical studies has been to validate anosmia as an early symptom of COVID-19 infection and to correlate the extent of olfactory sensitivity loss with the severity of global clinical manifestations. The rationale has been supported by data on the frequency of smell/taste loss in severe COVID-19 by objective testing, as reported by Mazzatenta et al. This study demonstrates that 95% of hospitalized individuals affected by severe COVID-19 reported smell dysfunction, while 47% were also affected by taste dysfunction. Furthermore, the majority of COVID-19 patients reported severe anosmia and the severity of the olfactory impairment correlate with symptom onset and hospitalization. This suggests that hyposmia, severe hyposmia, and anosmia may relate directly to infection severity and neurological damage. The results were strengthened by evaluation of taste and smell in either positive controls (subjects affected by chronic inflammatory diseases) or negative controls (healthy subjects). The test consisted in a logarithmic scale of n-butanol concentration to assess by positive answer: normosmia, hyposmia, severe hyposmia, or anosmia. The white odorless vial is the test's negative control. Gustatory test consists in a disposable two-item suprathreshold taste test (0.5 g/ml sucrose and 0.5 g/ml sodium chloride). Related to taste dysfunctions dysgeusia, xerostomia, and oral mucosal lesions are reported as the main oral symptoms of COVID-19 as reviewed in Lin et al. with the severity of COVID-19, therefore representing, in addition to olfactory dysfunction, a warning sign. A potential mechanism of dysgeusia may be related to taste buds dysfunction. Taste buds express ACE2, the receptor that the virus uses to infect the cells. In addition, or in alternative, dysgeusia may be a complication of an infection in the central or the peripheral nervous system. A similar mechanism is reported for Xerostomia and oral mucosal lesions, as the putative entry factors for SARS-CoV-2 are ACE2 and/or TMPRSS2, which are predominantly expressed in the salivary glands and oral epithelial cells. Another important issue is the duration of this early clinical manifestation after recovery from infection, especially in view of the high incidence of neurological manifestations in the long-term COVID sequelae. Regarding the relationship between long term COVID and loss of smell, the earliest article of the issue (2020) by Lechien et al. reports that among 88 affected by anosmia, 79.5% of patients recovered normal smell sense within 2 months, while for the remaining 21% olfaction required more time to recover. This is one of the first published evidence anticipating that we were dealing with a disease with an unusual multifaceted course, leaving behind signatures not easy to resolve. Viruses possess the most diverse ways to colonize the organism. An issue raised by smell/taste loss in COVID-19 patients is how SARS-CoV-2 reaches the brain regions involved in the healthy function of these senses. The contribution by Bilinska et al. consider that in spite of the preferential candidate route represented by the olfactory nerve, the virus entry receptor, i.e., angiotensin converting enzyme 2 (ACE2) is not or only poorly expressed in its neurons (Butowt et al., 2021). On the other hand, many peripheral processes of the nervus terminalis innervate the olfactory epithelium, and the central processes of some of these neurons reach various targets in the forebrain, and are in direct contact with the cerebrospinal fluid (Bilinska et al.). This study, conducted in mice, demonstrate that ACE2 is expressed in a small percentage of nervus terminalis neurons positive for choline acetyltransferase (CHAT) and in the majority of those showing immunoreactivity for gonadotropin-releasing hormone. Although indirect, these data indicate nervus terminalis as a plausible alternative for neuro-invasion of SARS-CoV-2, from the nose to the brain. Of interest, odontocetes, like dolphin, are highly susceptible to brain infection *via* nervus terminalis (Oelschläger et al., 1987; Damas et al., 2020).

It is impressive to see how quickly the scientific community has mobilized to study the virus and its effects, even as the pandemic continues to evolve. The contributions to this Frontiers topic demonstrate the breadth of research being conducted on COVID-19 and its neurological impact, from studies on the mechanisms of viral invasion to clinical observations of patients experiencing cognitive symptoms. As we continue to navigate this ongoing pandemic, it is crucial to remain vigilant in monitoring and addressing the long-term effects of COVID-19, particularly on cognitive function. The insights gained from ongoing research will undoubtedly prove invaluable in the development of effective treatments and therapies for those affected by this devastating virus.

## Author contributions

MM, AM, and NO conceived the outline of the Research Topic and wrote the editorial. All authors contributed to the article and approved the submitted version.
